# Clinicopathological evaluation of non-parasitic dermatoses in canines

**DOI:** 10.14202/vetworld.2015.1346-1350

**Published:** 2015-11-24

**Authors:** M. J. Sindha, B. J. Trangadia, P. D. Vihol, R. S. Parmar, B. V. Patel

**Affiliations:** 1 Animal Disease Diagnostic Laboratory, Amul Dairy, Mogar, Gujarat, India; 2 Department of Veterinary Pathology, College of Veterinary Science & Animal Husbandry, Navsari Agricultural University, Navasari, Gujarat, India; 3 Poultry Complex, College of Veterinary Science & Animal Husbandry, Anand Agricultural University, Anand, Gujarat, India; 4 Cambay Satellite Dairy, Amul Dairy,Undel, Gujarat, India

**Keywords:** bacterial, canines, fungal, non-parasitic dermatoses

## Abstract

**Aim:**

The present study has been carried out to detect non-parasitic dermatoses in canines brought at the Nandini Veterinary Hospital, Surat.

**Materials and Methods:**

The current investigation was carried out on skin scrapping, skin biopsy specimens, blood, and serum samples of 210 freshly registered cases of dogs with dermatological afflictions. Dogs found healthy on clinical examination were used as control animals (n=15). The incidence of non-parasitic dermatoses has been recorded as per age, breed, and sex of dogs. For bacterial isolation, the pus/exudates samples were collected from 40 cases of pyoderma and streaked onto brain-heart infusion agar while 13 skin scrapping samples were inoculated on Sabouraud’s dextrose agar with chloramphenicol for isolation of fungi. The organisms were identified on the basis of gross and microscopic observation of cultural growth on media. The blood and sera samples were also collected to note alteration in hematology and biochemical parameters, respectively. Tissue samples from lesions were collected and subsequently preserved in 10% neutral buffered formalin for histopathology.

**Results:**

Out of 210 cases of dermatoses, 60 cases were of non-parasitic dermatoses, i.e., 28.57%. Of these, bacterial skin infections (pyoderma) were found to be the predominant at 80.00%, followed by other non-parasitic dermatological disorders, i.e., 11.67% and fungal skin infection, i.e., 8.33%. The dogs belonging to age group 1-3 years showed greater susceptibility to non-parasitic dermatological conditions. Breed wise incidence of pyoderma was found more in the Pomeranian breed (20.83%), whereas fungal skin affections were found to be higher in mongrel breed (60.00% and 42.86%, respectively). Male dogs showed greater involvement in bacterial, fungal, and other non-parasitic dermatoses. Bacteriological culture examination of 40 pus swabs resulted in the growth of 39 bacterial isolates. Mycological culture of skin scrapings from 13 suspected cases of fungal dermatoses resulted in the recovery of five fungal isolates. Hematological and serum biochemical parameters revealed a significant difference in all cases of non-parasitic dermatoses.Histopathological study revealed characteristic changes like infiltration of neutrophils with perifolliculitis, hyperkeratosis, and rafts of acantholytic cells. Histochemical staining revealed purple or magenta color fungal elements.

**Conclusion:**

Based on current experiment it has been concluded that among non-parasitic dermatoses bacterial and fungal skin infections are the main ailments, followed by nutritional and other causes in adult and male dogs which can be diagnosed by cultural inoculation, microscopic examination of skin scrapings, and dermatohistopathology along with hematology and biochemistry.

## Introduction

Dogs are members of the order Carnivora, a group of mammals with origin in the tertiary era, about 55 million years ago [1]. The strong bonding between dog owners and pet dogs leads to concern about health and well-being of their pets and, therefore, the affected dogs are often brought to veterinary clinics for early diagnosis and treatment. Skin is the largest organ and outermost integument of the body and, therefore, is exposed to the adversities of the environment, allergens, pathogenic organisms and various toxic substances. “Dermatitis” is described as an inflammatory condition of skin regardless of the cause.

In small animal clinics, dermatological disorders constitute a majority of cases and are estimated to range between 12% and 75% as the chief or concurrent owner complaint [2,3]. Almost every dermatological disease of the dog can have bacterial pyoderma as a component [4]. There are so many agents that cause dermatitis including bacteria, fungi, yeasts and also other factors such as age, season and inadequate or unbalanced nutrition. The diagnosis can be made on the basis of history, physical examination, hematobiochemical findings, skin scrapping examination, cultural isolation of pathogens using various selective, and enrichment media from clinical specimens. Skin biopsies can be collected for histopathological and histochemical examination.

The present study was planned with objectives, to study the pattern and distribution of the skin lesions, laboratory analysis of blood and biochemical parameters, examination of skin scrapings to elucidate the effects of non-parasitic dermatoses and pathomorphological and histochemical evaluation of skin sections to assess changes in skin biopsies in selected cases.

## Materials and Methods

The current investigation was carried out on skin scrapping, skin biopsy specimens, blood and serum samples of 210 freshly registered cases of dogs with dermatological affections. Out of 210 cases of dermatoses, 60 cases were of non-parasitic dermatoses, i.e., 28.57%. Of these, bacterial skin infections (pyoderma) were found to be the predominant at 80.00%, followed by other non-parasitic dermatological disorders, i.e., 11.67% and fungal skin infection, i.e., 8.33%. Those animals found healthy on clinical examination were used as a control, and the hematobiochemical values of dogs with non-parasitic dermatosis were compared with them (n=15).

### Ethical approval

The present experiment has been carried out on apparently ail animals and include no any clinical trials on animals, further the author have taken informed consent of the pet owners.

### Study area

The present investigation was carried out on dogs brought at the Nandini Veterinary Hospital, Surat. Hemato-biochemical analyses were carried out in the Pathology Laboratory of Nandini Hospital, Surat. Histopathological and histochemical staining work was carried out in the Department of Pathology, College of Veterinary Science and Animal Husbandry, while the estimation of zinc was carried out in the Department of Agricultural Chemistry and Soil Science, NAU, Navsari.

### Collection of materials

Skin scrapping was collected from superficial and deep lesions as per established method [5,6] following strict aseptic measure in a test tube containing 10% of KOH and processed it for microscopic examination. Blood samples for evaluation of hematological parameters *viz*. hemoglobin (Hb) (g%), packed cell volume (PCV) (%), total leukocyte count (TLC) (10^3^/cumm), total erythrocyte count (TEC) (10^6^/cumm), and differential leukocyte count (%), were collected from jugular vein in a glass vials containing ethylenediaminetetraacetic acid and sera samples for biochemical parameters such as total protein (g/dl), albumin (g/dl), globulin (g/dl), A/G ratio, cholesterol (mg/dl), and zinc (µmol/L) were separated after clotting of blood by means of centrifugation at 500 G for 10 min with 0.1% methiolate asa preservative. The sample for bacteriological culture were collected as per sampling technique described by previous researchers [7] and inoculated on brain-heart infusion agar for primary isolation of bacteria, and Gram’s staining was performed for morphological characteristics of organism. For mycological culture and isolation, skin scales and epilated hairs were cultured on the slant containing Sabouraund’s dextrose agar with chloramphenicol at 37°C and examined at every 4-6 days up to 3-4 weeks for fungal growth. The gross appearance was on the basis of colonial growth on the test tube. For microscopic morphological examination, the slides containing isolates were stained with lectophenol cotton blue/Narayan stain and the presence of hyphae, spores were recorded as per the suitable method [8]. Collection of skin biopsy specimens were performed after locally anesthetizing the site (2% lidocain + epinephrine) using 3 mm circular punch at a depth of 2 mm in 10% neutral buffer formalin and subsequently stained with H and E stain [9].

### Statistical analysis

The data generated on hematological, biochemical, and trace mineral observations were subjected to statistical analysis for test of significance by applying appropriate statistical methods [10].

## Results and Discussions

**Figure-1:**
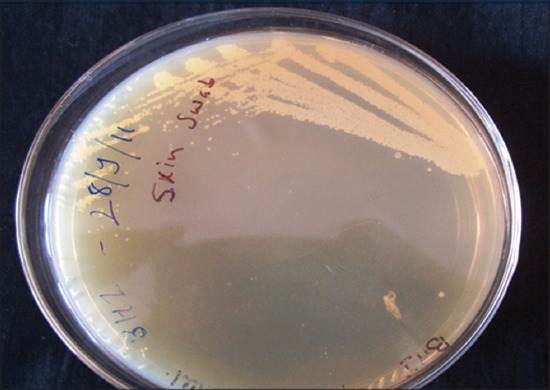
Dew drop like colonies of staphylococcus on BHI agar.

**Figure-2:**
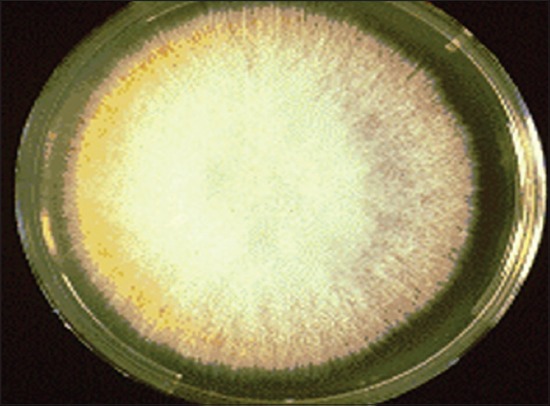
On Sabouraud’s Dextrose Agar Microsporum canis produce white cottony to wooly appearing colony.

In present study, after screening of 210 dogs with dermal affection 60 cases were found of non-parasitic dermatoses, out of theses 60 cases bacteria was found as an etiological agent in 48 cases (80%) and fungal infection was reported in 5 cases (8.33%). The highest incidence of both this condition was observed in the dogs of 1-3 years age group. Similar observation was made by other investigators [11-13] while some researcher reported the highest incidence of bacterial dermatitis in the dogs below 1 year of age (41.66%) [14]. When we considered breed, bacterial dermatoses (10 out of 48 cases) was the highest in Pomeranian whereas mycological affection (3 out of 5 cases) was maximum in mongrel dogs. High incidence of bacterial skin affection in long haired breeds of dogs was also reported by other researchers [12,15]. In current investigation, involvement of male dogs in bacterial and fungal dermatoses was observed to be more which was in agreement with a previous observation [15-17]. On bacteriological culture inoculation of 40 pus swabs resulted in the recovery of 39 bacterial isolates, out of which 36 isolates were of *Staphylococci* spp. appearing as a dew drop like colonies (92.30%) (Figure-1). Many workers have emphasized the role of *Staphylococcus*bacteria in producing pyogenic skin infections due to various exotoxins released by this organism [16,18]. Some investigators classified *Staphylococci* induced pyoderma into the surface (13.6%), superficial (66%) and deep pyoderma (20.5%) [19]. The observation made by some researcher suggests that obesed dogs and dogs of the pug-nosed breed are frequently affected by bacterial pyoderma in skin folds on their face, lips and vulva [20]. The inherent resistance of *Staphylococci* limits the usefulness of some medication like tetracyclines and simple penicillins [21]. On inoculation of total 13 skin scrapping specimens suspected for fungal infection, only 5 (38.16%) yielded isolates on mycological culture examination (Figure-2). Similar finding was obtained by earlier investigators[17] with only 10% positive cultures out of 8349 inoculated. Some researchers [22] reported that *Microsporum canis* and *Trichophyton mentagrophytes* infection in dogs was 4.4% and 2.2%, respectively while other [23] isolated *Penicillium* spp. (19.80%) and *Aspergillus* spp. (26.73%). The *Trichophyton* spp. (23%) was also frequently isolated along with *Microsporum* spp. (3%) and *Epidermophyton* spp. (1%) [24].The sample size of skin scrapings under this study was too small to draw conclusions about the result.

Hematological parameters in current study showed significant variation. The average values of Hb, PCV, and TEC were significantly lower in bacterial, fungal and other non-parasitic dermatological conditions when compared to control (Table-1). These findings were in agreement with previous results [25]. This decreased might be due to the reduced appetite and blood loss from scratching and inflammation while TLC values were found significantly higher in all cases of non-parasitic dermatoses as compared with control (Table-1). The leukocytosis could have resulted from toxins released due to tissue damage or necrosis produced by inflammation or from secondary bacterial infection. The values of neutrophils were found significantly higher in cases of bacterial skin diseases and significantly lower in cases of fungal skin diseases whereas blood lymphocytes showed vis-a-vis results when compared with control (Table-2). Neutrophilia in bacterial dermatitis might be due to mobilization of marginal and bone marrow granulocytic pool, and lymphocytosis as observed in fungal dermatitis could be due to persistent antigenic stimulation by chronic infection or inflammatory reaction produced by fungal infection [26]. The values of eosinophils were significantly higher in cases of fungal and other non-parasitic dermatological conditions with no significant difference in bacterial skin diseases when compared with control in contrast the values of monocytes were significantly lower in cases of bacterial skin diseases with the absence of basophils in peripheral blood (Table-2). The average values of serum biochemical parameters were compared with mean of corresponding normal (control group, n=15) dogs. The parameters such as serum globulin, A/G ratio in bacterial, fungal, and other non-parasitic dermatological conditions showed a significant difference when compared with normal control group, whereas other parameters were non-significant (Table-3). Significant increase in globulin with decreased A/G ratio as observed in the current study was also observed by previous researchers [25].

**Table-1:** Hematological profile (Hb, PCV, TEC and TLC) of dogs with non-parasitic dermatoses.

Groups	Parameters (mean±SE)				

	N	Hb (g%)	PCV (%)	TEC (10^6^/cumm)	TLC (10^3^/cumm)
Control	15	12.32±0.11	36.23±0.11	6.10±0.03	9.29±0.07
Bacterial skin infection	28	09.80±0.34**	29.40±1.03**	4.90±0.17**	14.21±0.18**
Fungal skin infection	05	09.97±0.36**	29.92±1.08**	4.99±0.18**	14.70±0.20**
Other non-parasitic dermatological conditions	07	08.98±0.21**	30.30±0.60**	5.05±0.10**	14.06±0.28**

** Highly significant at p≤0.01, with reference to control, N=Number of observations, NS=Non significant, Hb=Hemoglobin, PCV=Packed cell volume, TEC=Total erythrocyte count, TLC=Total leukocyte count, SE=Standard error

**Table-2:** DLC profile for non-parasitic dermatoses in canines.

Groups	N	DLC (%) (mean±SE)				

		N	L	E	M	B
Control	15	67.33±0.23	26.73±0.23	2.27±0.25	3.53±0.13	0.20±0.11
Bacterial skin infection	28	76.59±0.61*	18.23±0.57*	2.64±0.14*	2.50±0.16*	0.00±0.00
Fungal skin infection	05	54.27±0.58*	37.00±1.61*	4.73±0.18*	3.73±0.23*	0.00±0.00
Other non-parasitic dermatological conditions	07	65.77±1.27*	27.61±1.26*	3.08±0.24*	3.54±0.18*	0.00±0.00

* Significant at p≤0.05, with reference to control, N=Number of observations, NS=Non significant, DLC=Differential leukocyte count, SE=Standard error, N=Neutrophils, L=Lymphocytes, M=Monocytes, E=Eosinophils, B=Basophils

**Table-3:** Serum biochemical profile of non-parasitic dermatoses in canines.

Groups	N	Parameters			

		Total protein (g/dl)	Albumin (g/dl)	Globulin (g/dl)	A/G ratio
Control	15	7.36±0.10	3.43±0.05	3.93±0.13	0.89±0.04
Bacterial skin infection	28	7.56±0.05	3.31±0.03	4.25±0.06*	0.78±0.02*
Fungal skin infection	05	7.69±0.13	3.33±0.10	4.36±0.17*	0.77±0.05*
Other non-parasitic dermatological condition	07	7.55±0.03	3.32±0.04	4.23±0.04*	0.78±0.01*
Total	55				

**Parameters**		**Mean±SE**			

		**Control**		**Treatment**	

Cholesterol (mg/dl)		183.33±2.31 (N=15)		190.40±2.97 (N=07)	
Zinc (µmol/L)		13.81±0.71 (N=05)		13.01±0.53 (N=05)	

* Significant at p≤0.05, with reference to control, N=Number of observations, NS=Non significant, SE=Standard error, A/G=Albumin globulin ratio

**Figure-3:**
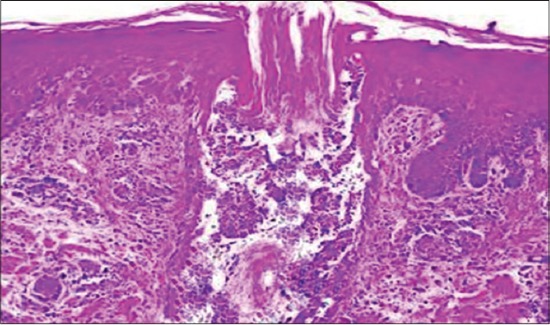
Perifolliculitis with infiltration of inflammatory cells (H & E, 100 X).

**Figure-4:**
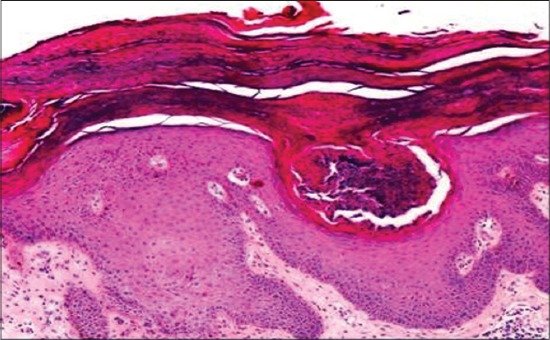
Skin biopsy sample from zinc responsive dermatoses revealed Parakeratosis (H & E, 400X).

**Figure-5:**
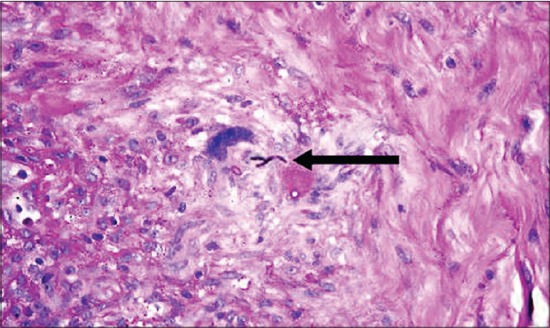
PAS staining of skin showing fungal hyphae- arrow (400X).

The histopathological alterations observed in the biopsy specimens from pyoderma cases revealed mainly infiltration of neutrophils with perifolliculitis (Figure-3). Whereas biopsy from lesions of infected callus (pressure point pyoderma) revealed perifolliculitis, hyperkeratosis, and rafts of acantholytic cells. Sections of skin infected with fungi showed fungal spores and hyphae along with other pronounced histological changes such as hyperkeratosis, acanthosis, and follicular changes. Biopsy specimen from zinc responsive dermatoses revealed characteristic change of parakeratosis (Figure-4). Infiltration of these cells in cases of bacterial infections suggests immunological and inflammatory response of body defense mechanisms [11]. Mechanical disruption of the stratum corneum appears to be important in facilitating penetration of dermatophytes and might be responsible for inflammatory and histological changes in the skin. The skin sections from the cases of fungal skin infections were stained with the periodic acid-Schiff stain which revealed purple or magenta colored fungal hyphae (Figure-5) which concur with the findings of Kumar and Kiernan[27].

## Conclusion

Based on current experiment it has been concluded that among non-parasitic dermatoses bacterial and fungal skin infections are the main ailments, followed by nutritional and other causes in adult and male dogs which can be diagnosed by cultural inoculation, microscopic examination of skin scrapings, and dermatohistopathology along with hematology and biochemistry.

## Authors’ Contributions

MJS perform the study under the guidance of BJT and PDV. MJS and BVP perform the laboratory investigation of the samples. MJS and RSP drafted the final manuscript. The final manuscript was read and approved by all the authors.
